# Connecting Gas-Phase Computational Chemistry to Condensed Phase Kinetic Modeling: The State-of-the-Art

**DOI:** 10.3390/polym13183027

**Published:** 2021-09-07

**Authors:** Mariya Edeleva, Paul H.M. Van Steenberge, Maarten K. Sabbe, Dagmar R. D’hooge

**Affiliations:** 1 Laboratory for Chemical Technology (LCT), Ghent University, Technologiepark 125, 9052 Zwijnaarde, Belgium; Paul.VanSteenberge@UGent.be (P.H.M.V.S.); Maarten.Sabbe@UGent.be (M.K.S.); 2 Industrial Catalysis and Adsorption Technology (INCAT), Ghent University, Valentin Vaerwyckweg 1, 9000 Ghent, Belgium; 3 Centre for Textile Science and Engineering (CTSE), Ghent University, Technologiepark 70a, 9052 Zwijnaarde, Belgium

**Keywords:** ab initio, chain growth, step growth, rate coefficients, solvation models

## Abstract

In recent decades, quantum chemical calculations (QCC) have increased in accuracy, not only providing the ranking of chemical reactivities and energy barriers (e.g., for optimal selectivities) but also delivering more reliable equilibrium and (intrinsic/chemical) rate coefficients. This increased reliability of kinetic parameters is relevant to support the predictive character of kinetic modeling studies that are addressing actual concentration changes during chemical processes, taking into account competitive reactions and mixing heterogeneities. In the present contribution, guidelines are formulated on how to bridge the fields of computational chemistry and chemical kinetics. It is explained how condensed phase systems can be described based on conventional gas phase computational chemistry calculations. Case studies are included on polymerization kinetics, considering free and controlled radical polymerization, ionic polymerization, and polymer degradation. It is also illustrated how QCC can be directly linked to material properties.

## 1. Introduction

Polymers are typically produced in large-scale industrial reactors, yielding (i) low-cost bulk commodity materials for e.g., household goods, including packaging, construction materials, and heat insulation, as well as (ii) high-added value polymers, which are typically fine chemicals and pharmaceuticals for high-tech niche applications e.g., biomedical devices, medicines, and electronics [1,2,3,4,5,6,7,8,9,10,11]. These polymers can be produced through two main mechanisms, i.e., chain-growth polymerization and step-growth polymerization, each involving numerous elementary reaction steps. In the former mechanism, one has in essence initiation, chain initiation, and many propagations, until termination creates “dead” polymer molecules on a very short time scale ((m)second scale) [12]. In the latter mechanism, functional groups are combined step by a step, and a gradual transition from monomer to dimer to *n*-mer takes place [13].

Due to the complex interplay of the elementary reactions in the given polymerization process, the link between the initial process parameters and the molecular and material properties of the final polymers is not straightforward. Thus, for optimal performance of industrial polymerization processes, it is advised to perform model-guided process design, which relates the changes in polymerization kinetics through variations of physical parameters (e.g., temperature, pressure, reactant concentrations, and solvent choice) with macromolecular properties (e.g., viscosity and strength). 

**Figure 1:**
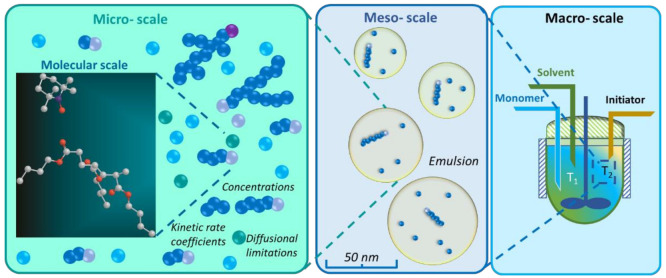
Scales in polymerization, leading to different calculation levels. A case is shown with particles present so that a meso-scale is recommended.

From an engineering point of view and as shown in Figure 1, three levels for the length-scale-based description of polymerization processes exist: the (1) micro-scale (with as sub-scale the molecular scale), (2) meso-scale, and (3) macro-scale [14]. Most of the focus in engineering studies has been on the micro-scale, which is defined as the scale at which (molar) concentrations can be defined. The key input parameters for that scale are typically Arrhenius and diffusion parameters, which are determined based on data fitting to experiments [15,16]. This emphasis on the micro-scale is also clear from an inspection of more recent reviews with only a few works addressing either the molecular or meso/macro-scale [17,18,19,20,21]. At the industrial or macro-scale, polymerization reactors are frequently inhomogeneous, leading to the formation of zone-dependent bulk concentrations and/or polymerization temperatures, resulting in a high level of product inhomogeneity between the zones (or thus lower length scale). The meso-scale is only relevant if particulate systems are considered with e.g., the recent work of Marien et al. [22] displaying that the evolution of the chain length and particle size distribution is coupled in miniemulsion polymerization at which the “reactor” size is very low (below 200 nm).

From a more fundamental point of view, the molecular scale should be treated with care and properly connected with the engineering micro-scale. In other words, the detailed description of polymerization processes requires significant chemistry driven efforts, as the reaction mechanisms contain a large number of elementary reactions involving likely both uni- and macromolecular species and for sure involving main and side reactions. The associated reactivities may significantly depend on the chain length; thus, ideally, for each macromolecular species type, a complete chain length distribution has to be taken into account [23,24,25,26,27,28,29,30]. Additional complexity arises for those processes with multiple reactive species. For example, in the radical polymerization of acrylates, two main types of active species exist, namely end-chain radicals and mid-chain radicals with the former much more reactive than the latter. Even more complex is for instance the radical polymerization of vinyl chloride with ca. 10 radical types and multiple reactive sites in the polymer backbone that need to be accounted for in an advanced kinetic model also aiming at the understanding of poly(vinyl chloride) stability [14,29,31].

The occurrence of side (or secondary) reactions can largely influence the molecular structure of the polymer molecules, e.g., the formation of head-to-head effects, unsaturations, and branches, therefore altering the macroscopic behavior. For example, at high monomer conversion, chain transfer to polymer or intermolecular chain transfer can take place and long-chain branches (LCBs) can be formed, whereas short-chain branches (SCBs) can be created through shift reactions, including backbiting. Focusing again on polyacrylates, a more branched structured has a much more complex viscosity behavior as a function of shear rate during subsequent processing in a final product. If a copolymerization is considered, the compositional drift may also affect the molecular structure and one can require reactivity models acknowledging penultimate and thus monomer neighboring effects. The reactivities for these secondary reactions are unfortunately often difficult, sometimes even impossible to access via experimental techniques, due to the inability to decouple them from other primary and secondary reactions involved in the polymerization mechanism [20,32].

To help to solve the previous challenge for secondary reactions in the large field of chemical processes, quantum chemical calculations (QCC) can be applied. In general, such calculations are performed in the gas phase, whereas most of the experimental data are available in the condensed phase, which is especially the case for polymerization reactions. If QCC are used to access the rate coefficients (so-called *k_(chem)_* values) that are hard to measure experimentally, the extrapolated solution-phase computational data are of interest, as they can be directly applied for the construction of the actual kinetic models in view of (polymerization) process control. Thus, a number of QCC approaches have been developed that take solvation into account. Reliability has always been an issue for QCC, but more recent developments in several research groups have provided a large set of more reliable absolute *k* values that can plugged into kinetic models. In any case, QCC is rather reliable in predicting relative reactivities, facilitating the interpretation of reaction pathways.

In this review, the most important QCC approaches to account for solvent effects are described in general terms. A roadmap is provided starting from a concise overview of gas phase GCC approaches to then tackle the solvation models to allow for a transition to the condensed phase. Specific focus is on QCC for (molecular scale) rate coefficients and the additional conceptual treatments needed to address specifically polymerization reactions, aiming at a reader that is less familiar with GCC but is active in the field of polymer (reaction) engineering or chemistry. It is also explained how the QCC-based rate coefficients need to be combined with actual polymerization kinetic models that are dealing with larger length scales. This is further exemplified by including four case studies from the field of polymerization kinetics but also one in a broader frame of material property prediction. 

## 2. Gas Phase QCC Approaches

Quantum mechanical approaches rely on the solution of the Schrödinger Equation (1), which describes molecules in terms of nuclei and electron interactions:

(1) $$\hat{H}\psi \left(x,t\right)=E\psi \left(x,t\right)$$

in which $\hat{H}$ is the Hamiltonian operator, *ψ(x,t)* is the system’s wave function (eigenvector), and *E* is the total energy of the system (eigenvalue). In principle, the solution of the Schrödinger equation (SE) provides the complete description of the molecular structure and energy. However, the exact solution is available only for the simplest one-electron system (e.g., the hydrogen atom), as the electron–electron interaction term makes it impossible to construct an analytical solution for complex molecules. Thus, the general exact wave functions remain unknown. It should be mentioned that the time dependence in Equation (1) is typically neglected, and only the stationary SE is solved.

QCC explore various pathways toward an approximate solution for the wave functions, among which the Born–Oppenheimer approximation [18] is the most common. The latter assumes that since the nuclei are much heavier, their motions are negligible at the time scales of the electron motions. A further assumption is made that the electrons are responding instantaneously to changes in the nuclear configuration and, thus, nuclei move in the mean electron field. By these means, the so-called electronic Schrödinger equation (ESE) is introduced, describing the motion of *N* electrons in the field of *M* point charges generated by the nuclei, and the wave function explicitly depends only on the electronic coordinates [33].

**Figure 2:**
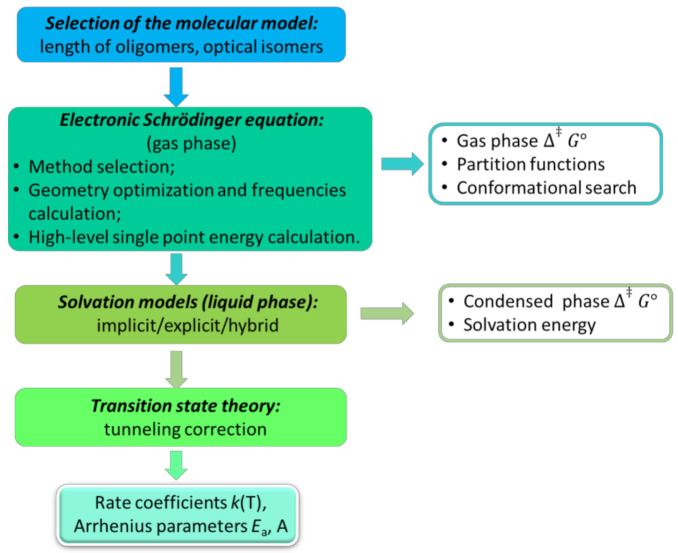
General algorithm for QCC to finally retrieve kinetic parameters; case of polymerization chemistry.

Practically, more assumptions are needed than just applying ESE. The successful prediction of the kinetic parameters is directly linked to an overall QCC methodology of which the most important steps are highlighted in Figure 2. The first step is the selection of an appropriate molecular model, which for reactions involving macrospecies is of high importance. One has in reality polymer chains containing thousands of monomer units, which is impossible for QCC methods to grasp. This leads to the approximation of the polymer chains by oligomers with a chain length of two, three, or longer depending on the computational costs. The specific optical isomers—in case of polymer chains, the tacticity—should also be (ideally) considered in this first step, and the most probable (from a thermodynamic of kinetic perspective) should be employed for the further investigations. In the next step, the electronic energy and (approximate) wave functions are calculated, and the geometry is optimized for the reactants, transition state, and products in the gas phase, providing the thermodynamic parameters in this phase. If the reaction in condensed phase is considered, then the research should select in a third step the appropriate solvation model. The above-mentioned steps provide the input for the transition state theory (TST) calculations, which, in the end, lead to the prediction of the kinetic rate coefficients at the selected temperatures and, hence, the Arrhenius parameters. 

Notably, the research on approximating approaches to solve the SE is vast. The reader is referred to the following contributions to obtain a broader scope [34,35,36]. In the following sections, the aforementioned steps in Figure 2 are concisely discussed, paying special attention to the solvation models. 

## 3. Gas Phase Computational Chemistry Tools

A variety of gas phase computational chemistry tools exists. In the present section, a differentiation is made between wave-function-based methods, density functional theory (DFT) calculations, and transition state theory TST; hence, a further elaboration is presented regarding the individual blocks in Figure 2. Semi-empirical methods are addressed as well.

### 3.1. Wave-Function Based Calculations

For the first instance, the wave function is approximated by the linear combination of one-electron molecular orbitals *ψ_i_(x,y,z)*, which are expressed as a linear combination of one-electron atomic orbitals $\varphi_{\mu }$, which is the so-called ‘basis set’:

(2) $$\psi_{i}=\overset{n}{\underset{\mu =1}{\sum }}C_{\mu i}\cdot \varphi_{\mu }$$

If the wave function is constructed, the expansion coefficients (*C_µi_*) are optimized by minimization of the system’s energy. For example, in the Hartree–Fock method (HF) [34,37,38], the wave function is formed as the Slater determinant. Among all the *wave-function-based* approaches, HF is only the starting point, as the approximation that a particular electron moves in the averaged field generated by all other electrons, rather than interacting with all other electrons individually, does not lead to accurate electronic energies. More advanced methods, e.g., the perturbation theory in the formalism of Møller–Plesset (MP), configuration interaction (CI) [39], and coupled-cluster approaches (CC) [40], go beyond this approximation by including ‘correlation’ between the electrons via the inclusion of excited-state wave functions at the cost of significantly increased computational demands [41].

### 3.2. Density Functional Theory

DFT is based on the Hohenberg–Kohn theorem [42] stating that the ground-state electronic energy can be calculated from the ground-state electron density so that the wave function does not need to be evaluated. DFT introduces a one-particle functional that contains all the many-body effects, making it possible to compute in principle the exact ground-state energy from the electron density. Although it has been shown that such a functional exists, its form is in general unknown. Thus, the variations of the functionals that are used for the calculations represent some sort of approximation. Thus, it is constructed on the basis of one-electron functions that form a basis set (which is similar to the wave-function-based method). 

The basis set significantly influences the accuracy of the calculations; thus, its selection is crucial for the accurate prediction of the kinetic parameters. In a number of contributions, the calculation results with different basis sets have been compared [43,44,45]. Depending on the chemical system and type of the reaction calculated, different basis sets can provide more accurate results [46,47,48].

Due to the low computational demands of DFT, it is widely applicable for QCC for the chemical reactions involved in a polymerization mechanism. Furthermore, in contrast to most HF-based methods, DFT has no formal issues dealing with resonance-stabilized radicals, which is valuable if, e.g., radical polymerization processes are studied theoretically [49,50,51,52,53,54,55,56].

### 3.3. Semi-Empirical Methods

Wave-function-based and DFT methods are well-established for relatively small molecular models so that for polymers, the above-mentioned models treat three to five monomer–oligomer units. For the larger molecules, the computational cost can quickly become prohibitively high. One can compare e.g., the computational time required to optimize the geometry of polyacrylate model oligomers consisting of two and five monomeric units (DFT, B3LYP/6-311+G(d,p)) being 1 h and 11 h respectively, considering six CPUs in parallel (on a 2 × 18-core Intel Xeon Gold 6240 processor). For most methods that yield highly accurate energies, the computational effort will be higher and moreover scale worse with the molecular size.

To facilitate the calculations for larger molecules, especially polymers and bio-molecules, several semi-empirical theoretical methods have been developed. The overview of the semi-empirical methods was revised recently by Bryce et al. [57]. To mention, PM3, AM1, and MNDO are suitable to obtain the enthalpy of formation of chemical systems. Developed by Dewar et al., the MNDO [58] and AM1 [59] methods became a standard tool for both theoretical and experimental organic chemists. Later, Stewart et al. [60,61] proposed a mathematical reparameterization of the MNDO method called the PM3 method. In this method, single-atom parameters were obtained for C, N, H, O, F, S, P, Si, Cl, Al, I, and Br simultaneously, by fitting 400–500 experimental references. Generally, PM3 provides more accurate results than the AM1 method. However, most of these methods do not perform well to locate transition states or predict activation barriers.

Another method to mention is ONIOM [62]. In this method, a large molecular system is divided into several layers, which are treated at a different level of theory, leading to sufficient decrease in the computational demands [33,48]. Zhang et al. [63] demonstrated the cost-effectiveness of ONIOM calculations by comprehensive investigation of MMA homopolymerization on different levels of theory. The particular field of ONIOM calculations is catalysis and enzymatic catalysis, in which the active catalytic site is treated on the highest level of theory [64].

## 4. Solution Phase Computational Chemistry Tools

### 4.1. Transition State Theory

The connection between the kinetic parameters of the chemical reaction under investigation and the thermodynamic properties that can be derived from QCC is provided by transition state theory (TST), which is applicable not only for the gas phase [65]. TST links the reaction rates via microscopic partition functions and the reaction barrier at 0 K. This theory assumes a statistical equilibrium between the degrees of freedom of the reactants, products, and transition state. In conventional TST, the transition state structure is located at the maximum energy structure along the minimum energy pathway that connects the reactants and products. With the minimal number of optimized geometries (for the transition state and reactants), one can access the kinetic parameters of a particular reaction in the following way:

(3) $$k\left(T\right)=\kappa \frac{k_{B}T}{h}\frac{Q^{‡}}{\prod Q_{i}}c{}^{\circ}e^{-E^{‡}/RT}=\kappa \frac{k_{B}T}{h}ce^{-\Delta ‡G{}^{\circ}/RT}$$

in which κ is the transmission coefficient accounting for quantum tunneling (assumed to be a value of one in the simplest case), $k_{B}$ is the Boltzmann’s constant, *h* is the Planck’s constant; Δ^‡^*G°* is the standard Gibbs free energy difference between the reactants and the transition state, E^‡^ is the electronic energy difference between the reactants and the transition state; and *Q^‡^* and *Q_i_* are respectively the molecular partition function of the transition state and the reactant *i*. The molecularity of the reaction *m* should be taken into account by multiplication by $c{}^{\circ}={\left(P{}^{\circ}/RT\right)}^{1-m}$ to obtain the correct units in concentration terms.

The molecular partition functions form the link between the quantum mechanics and the thermodynamic properties. Typically, they are calculated assuming separability of the partition function into a translational term (Q_trans_), a rotational term (Q_rot_), a vibrational term (Q_vib_), and an electronic term (Q_elec_). The partition functions are usually calculated under the assumption that the reaction occurs in the gas phase and the reactants and products are ideal gases. The vibrational part is usually calculated based on the harmonic oscillator approximation, although explicitly considering internal rotations and other large-amplitude motions became common to obtain accurate gas-phase thermodynamics and kinetics.

Splitting the Gibbs free energy to enthalpic (*H*) and entropic (*S*) contributions and considering the classical temperature dependence of kinetic rate coefficients, one obtains the relation between the Arrhenius parameters and calculated thermodynamic functions of the transition state:

(4) $$k\left(T\right)=\frac{k_{B}T}{h}e^{-\frac{\Delta G^{‡}}{RT}}=\left(\frac{k_{B}T}{h}e^{\frac{\Delta S^{‡}}{R}}\right)e^{-\frac{\Delta H^{‡}}{RT}}=Ae^{-\frac{E_{a}}{RT}}$$

One of the TST assumptions is that the motion along the reaction coordinates can be expressed as a simple, classical translation. However, it is only valid for larger molecules for which the De Broglie wavelength associated with the molecular rearrangement in the transition state is small compared to the width of the reaction barrier. For reactions that involve smaller species, i.e., hydrogen transfer, the quantum effects are relatively important. Thus, a factor κ that accounts for the tunneling effect is introduced [66,67,68]. However, for the direct comparison of the calculated thermodynamic parameters and experimentally determined *E_a_*, the tunneling effect and the temperature dependence of $\frac{k_{B}T}{h}e^{\frac{\Delta S^{\neq }}{R}}$ are often neglected compared to $e^{-\frac{\Delta H^{\neq }}{RT}}$, which is a common yet rather crude approximation.

Notably, the contributions of Heuts et al. [69,70] give an excellent example of QCC of the (gas phase) propagation reaction of ethylene with the complete calculation algorithm, which can be followed in order to correctly predict the kinetic rate coefficients. Furthermore, evaluating the current set of data for gas-phase processes in general, for various types of chemical reactions (mono-, bimolecular, with or without radical species, etc.), different methods of QCC are now applicable, as the calculated predictions are closer to the experimentally measured values in the gas phase. Benchmark studies have been even performed to derive the best practices in the theoretical evaluation of the kinetic parameters. For example, Meier et al. [71] extensively discussed the performance of DFT methods with respect to accuracy and reliability. Another example is the work of Green et al. [72,73] in which different DFT and MP2 methods have been compared for alkoxy radical rearrangement and gas-phase hydrogen abstraction reactions. More advanced comparison of theoretically calculated and experimentally measured kinetic parameters for gas-phase radical addition/β-scission was more recently performed by Sabbe et al. [74].

Note that all QCC provide results at 0 K, whereas the experimental temperatures are much higher. Via TST, the rate coefficients can be calculated at any temperature, thus providing access to the Arrhenius parameters. In this aspect, the accuracy for the calculation of the partition functions plays a significant role. In particular, the correct accounting of the internal rotations can affect significantly the calculation results as put forward by Van Speybroeck et al. [75] and Vansteenkiste et al. [76]. In addition, pressure effects can also be tackled via QCC, which is important for the translation of micro-scale to macroscale simulations. Several examples are provided in the literature [77,78,79,80,81].

### 4.2. Solvation Models

**Figure 3:**
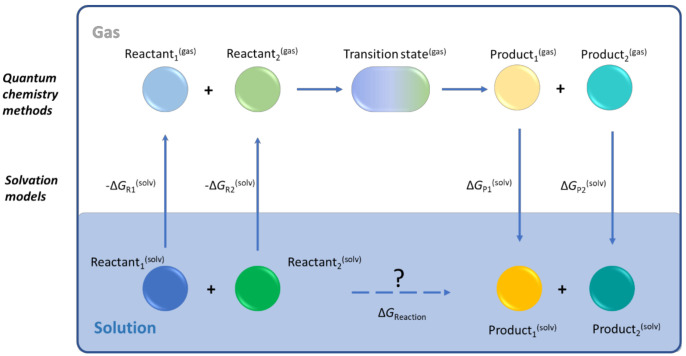
Thermodynamic cycle used to go from gas phase to condensed phase GCC.

QCC are conventionally performed in the gas phase, treating all the molecules as ideal gas components. To correct for the effect of the solvent, a solvation model is used in a thermodynamic cycle. It accounts for the desolvation of reactants and the solvation of the products of a chemical reaction, while the actual reaction step is still treated with QCC in the gas phase, as shown in Figure 3. More advanced methods also account for the influence of the solvent on the stabilization of a particular geometry of the TS. A number of solvation models allows for the correction of the gas-phase calculations toward the condensed phase. As explained in what follows, the models can be referred to as implicit, explicit, and hybrid.

#### 4.2.1. Implicit Models

The implicit solvation models treat the solvent as a continuum with certain properties. As these types of models are less computationally demanding, they are widely used for the description of the solute–solvent interactions. A number of authors reviewed the implicit models [82,83,84,85,86,87,88,89,90,91,92]; therefore, only a short description of the most common ones is provided here. A distinction is made between the Polarizable Continuum Model (PCM) [93,94,95,96], the *co*nductor-like *s*creening m*o*del (COSMO) [97,98,99], the Poisson–Boltzmann model (PB) [100,101,102,103,104], the generalized Born (GB) model [105], and the solvation model based on density (SMD) [106,107].

In PCM, the solvent is treated as a polarizable continuum medium that is characterized by its dielectric constant ε. The two most important characteristics of PCM are the use of a molecular cavity, which follows the real geometry of the system, and the surface charge distribution, which includes the polarization of the environment. PCM has the possibility of accounting for any shape and charge distribution. PCM is also characterized by ease of use and can be seen as a computationally cheap methodology that can be applied in systems in which short-range solute–solvent interactions play a major role.

COSMO has been developed in parallel to PCM [108,109,110,111] and has similar features; in particular, the condensed phase is modeled as a conductor with an infinitely large dielectric constant ε, which simplifies the electrostatics computations. COSMO includes non-electrostatic solute–solvent interactions as well, although it can give false predictions in case of specific interactions such as the formation of a complex with solvent molecules. Although COSMO is a common approach for accounting for solvent effects, it has been noted that this procedure may overestimate entropic factors [112]. To account for more sophisticated effects, advanced hybrid COSMO-RS methods, as described below, are typically employed.

The PB model can be applied to describe the electrostatic potential and equilibrium distribution of ions around molecules in solution, which makes it useful for the interactions of physiological processes and bioinformatics, polymer science, and electronics [92,100,113,114]. With the help of the Poisson–Boltzmann equation, the distribution of the electric potential in the solution can be described, and thus, the electrostatic interactions can be modeled. In polymer science, PB is typically applied for the quantum mechanics calculations of aqueous polymers, polyelectrolytes, and ionization processes [115,116,117]. The GB model [118] is an extension of the PB model, which relies on the (linearized) Poisson–Boltzmann equation. Due to its ability to predict charge distributions, it is widely applied for biomacromolecules [119,120].

In turn, the SMD model was introduced by Marenich et al. [106] in 2009. SMD is described as a universal continuum solvation model. By “universal”, the authors assume its applicability to any charged or uncharged solute in any solvent or liquid medium for which a few key descriptors are known. In this implicit model, the solvent is represented as a dielectric medium with surface tensions at the solute–solvent interface. The SMD model separates the fixed-concentration free energy of solvation into two components: (i) the bulk-electrostatic contribution and (ii) short-range interactions between the solute and solvent molecules in the first solvation shell. The first component is the bulk-electrostatic contribution arising from a self-consistent reaction field (SCRF) treatment. The SCRF treatment involves an integration of the nonhomogeneous-dielectric Poisson equation for bulk electrostatics in terms of the COSMO model. The second is the cavity–dispersion–solvent-structure (CDS) term, which accounts for the solvent-accessible surface areas (SASAs) of the individual atoms of the solute.

#### 4.2.2. Explicit Models

If an explicit solvation model is considered, the calculation of geometry and energy optimization is performed for a system with the solvent molecules explicitly included. Molecular dynamic calculations are commonly used as well for structure optimization here. For that, the concept of the “supermolecule” is introduced, which consists of a reactant/product and a surrounding solvent molecular ensemble. A supermolecule is made of an aggregate of the solute and a limited number of solvent molecules treated as an isolated single molecular system at the desired quantum chemical level. Depending on the accuracy, from one to 40 solvent molecules can be taken into account.

Some success has been achieved in the application of explicit solvent models to predict organic and inorganic reaction mechanisms [121,122,123], electrochemical phenomena [122,124,125,126,127,128], and pK_a_ values [129,130,131,132,133]. Such types of calculations predict well short-range effects such as hydrogen bonding but produce incorrect results for the long-range effects computation. Furthermore, due to the increased complexity of the system, i.e., reactant/product + several solvent molecules, the computational costs are high.

#### 4.2.3. Hybrid Models

To combine the strong sides of implicit and explicit solvation models, some hybrid approaches have been developed. Typically, such types of models treat first the solvation sphere explicitly while treating the surrounding solvent by an implicit model. Another approach is to account for the inner solvation shell by quantum mechanical models, and the outer solvation shell is covered classically. The latter approaches are the so-called QM/MM methods.

A realistic description of a chemical reaction in solution requires the sampling of all possible configurations of the solvent molecules around the solute, which can be achieved through molecular dynamics (MD) simulations. The reader is referred to previous work on the description of the principles of MD simulations as such [134]. For large systems, the computational costs are too high though. The efficiency of MD simulations of large solute–solvent systems can be greatly improved with a “multiscale” approach in which the solvent molecules in the first solvation shell are obtained using a quantum mechanical (QM) description, while the long-range effects are approximated by implicit solvation [135]. A more complex model uses the same QM description for the closest solvent molecules but describes the rest of the system explicitly with molecular mechanics (MM).

Originally, the systems studied with QM/MM were biomolecular proteins or other relatively rigid systems [136]. Nowadays, there is an increasing interest in extending the application of these QM/MM models to highly diffusive systems (e.g., homogeneous/heterogeneous catalysis and chemistry of solvated systems) [137,138,139,140,141,142,143]. Several QM/MM solvation models have been developed for this purpose, providing a similar level of accuracy [144]. Chemical reactions involving charged or zwitterionic intermediates are especially strongly affected by solvent. The intermediates are stabilized in polar protic solvents, while they are destabilized in apolar solvents, often yielding different mechanisms.

The extension of the COSMO model toward a hybrid model is COSMO-RS [109,145], which is the statistical thermodynamics theory based on COSMO polarization charge densities. This model overcomes many of the limitations of dielectric continuum models such as PCM and COSMO. As this model gives the possibility of treating mixtures at variable temperatures, it is widely applicable for chemical engineering as well as physical and medicinal chemistry. COSMO-RS may currently be considered the most accurate model for the prediction of solvation energies as well as the description of solvation effects for intermediates. The transition states provided by COSMO-RS can be seen as rather consistent as well.

#### 4.2.4. Advantages and Disadvantages

Each method of accounting for the solvent–solute interactions has certain advantages and disadvantages, as gathered in Table 1. Typically, a balance between the accuracy and the computational costs exists. If a solvation model is selected for a particular system, the user should keep in mind the specific features of the molecular model under investigation and the targeted accuracy. In certain general cases, application of the most simple models provides good qualitative agreement with the experimental data and reveals additional features of the specific reactions. For more advanced applications, e.g., prediction of the kinetic rate coefficients or revealing the effect of specific interactions, more complicated models should be employed. Notably, in the case study section (Section 6), it is illustrated how the application of solvation models influences the results of QCC in terms of agreement with the experimental data.

## 5. Connection of Lower-Scale Modeling with Higher-Scale Modeling

In TST, emphasis is on the calculations of the intrinsic rate coefficients (*k* values) at given temperatures. Thus, infinitely fast diffusion is assumed if one simply defines the related intrinsic rates in the next phase to describe concentration changes. For gas-phase kinetics, this can be reasonable, but for condensed-phase kinetics with high viscosities as in polymerization, this is not the case.

The most convenient way to account for this viscosity effect is to replace the intrinsic by apparent rate coefficients, the latter being a function of the intrinsic rate coefficients and the diffusivity of the reactive species involved. One of the most common approaches to evaluate the apparent reactivity is the so-called parallel encounter pair model, which assumes that molecules have to diffuse toward each other (*k*_+diff_) to form a so-called encounter pair before the actual reaction step (*k*_chem_) occurs. In general, all stages of polymerization (initiation, propagation, termination, …) are influenced by diffusional limitations. For a more detailed description, the reader is referred to following references [146,147,148,149,150].

An additional step toward more detailed kinetic modeling is the accounting of the non-isothermicity of the reactor. For example, most (radical) polymerization kinetic modeling studies focus on simplified (theoretical) isothermal conditions and/or consider only ballpark values for the Arrhenius parameters. In a recent study, Edeleva et al. [151] introduced the first kinetic Monte Carlo (*k*MC) model featuring an energy balance for a small-scale (two liter) perfectly mixed batch reactor with the explicit accounting of energy conservation in the presence of a cooling medium. As the reactor size was small and the solution conditions had been selected, perfect macro-mixing could be safely assumed.

However, the desire to model any reactor size requires at some point corrections for macro-scale variations. Note that also meso-scale variations could matter if one has particulate polymerization processes; however, in view of the scope of the present work, this scale is not considered, meaning that either the particles are sufficiently large to assume “bulk” reactor behavior or such particles are absent.

For the modeling of the macro-scale, two main approaches exist, i.e., compartment modeling and computational fluid dynamics (CFD). One of the more recent compartment modeling strategies to cover kinetics is the one of D’hooge et al. [152], as illustrated for atom transfer radical polymerization with electrodes, with conceptually similar calculations at meso-scale for droplet sizes in suspension polymerization in the work of Dompazis et al. [153]. The CFD-based methods have been recently reviewed by Drikakis et al. [154] and Pan et al. [155]. The contributions by Xie and Luo, who performed numerous studies of the polymerization reactors by means of hybrid CFD-based models, are notable as well [156,157].

In addition, for industrial application, one strives to unlock the possibility of fed/semi-batch reactor operation. In particular, Lemos and Pinto [158] have coupled *k*MC modeling with residence time distributions to model continuous stirred tank reactors in a hybrid analytical/stochastic manner. In the series of articles by Hutchinson et al., solution *k*MC models have been expanded toward semi-batch reactors with instantaneous addition of the reactants at predetermined times [159,160,161,162,163,164]. This method is efficient for non-starved feed conditions and for abundant chemical components. D’hooge et al. [165] have reported a similar *k*MC model for controlled radical polymerization (CRP) processes aiming at the production of “forced” gradient copolymers. On the deterministic front, Luo et al. [166] reported a reversible addition fragmentation transfer (RAFT) polymerization model accounting for inlet streams to the reactor to produce hyperbolic, parabolic, and linear gradient polymers.

For completeness, it is mentioned here that force-field modeling can contribute to filling the gap between atomic-level calculations and dynamics of the chemical systems on a higher scale. Among others, reactive force-field (ReaxFF) [167] can be mentioned. It combines a QCC approach for guidance to the atomic-level effects with empirical interatomic potentials within a bond-order formalism describing dynamic processes over longer time frames and on larger scales. This method is particularly useful for the calculations of the chemical reactions, as other force fields can produce less reliable results in the modelling of changes in atom connectivity, as bonds break and form through chemical reactions. In ReaxFF, connection-dependent terms in the force-field description are included, thus allowing for simulations of the chemical reactions. ReaxFF is not universally applicable, but the method has its merits for large-scale systems and calculations of a more qualitative nature. The special application of ReaxFF is the calculation of inter-phase events, making this method popular for biological processes [168,169], catalysis [170,171], and transport phenomena in energy storage applications [172,173].

In all the discussed models in this section, one still needs the intrinsic values. Sometimes, larger-scale phenomena fully mask the intrinsic effect (e.g., strong diffusional limitation on radical termination), but in general, the intrinsic rate coefficients have still impact. In other words, the reliability of the larger-scale models relies on these intrinsic values, again highlighting the need to further develop GCC and to connect GCC with larger-scale modeling studies.

## 6. Case Studies for Connection of Computational Chemistry and Kinetic Modeling

In the present section, it is explained that a correct GCC implementation is relevant to enable a correct description of four case studies on polymerization kinetics. It is assumed that no particles are present and no macro-scale effects are active. Hence, a micro-scale kinetic modeling approach is applied. A fifth case study is also included as well in which GCC is directly linked to material properties.

### 6.1. Case Study 1: Radical Polymerization

**Figure 4:**
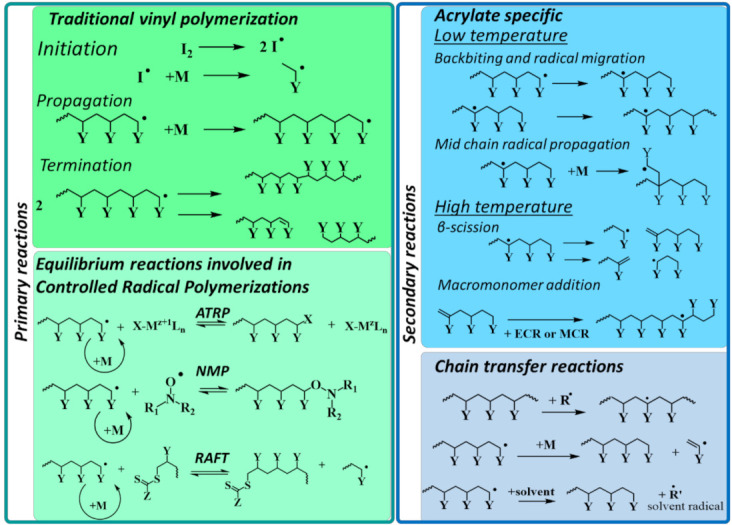
Primary and secondary reactions in free radical polymerization (FRP); specific focus is on the FRP of acrylates; also included are the extra reactions if one goes from FRP to controlled radical polymerization (CRP).

In general, the mechanism of free-radical polymerization (FRP) with vinyl monomers involves initiation, propagation, and termination reactions, as shown in Figure 4 (top left). For particular families of monomers, e.g., acrylates, a number of monomer-specific secondary reactions may occur as well (Figure 4; top right). Typically, the activation energies of the secondary reactions are sufficiently higher than the ones of the primary reactions, which decreases their occurrence at lower temperature but increases their relative importance upon temperature increase. Furthermore, as they occur simultaneously with the primary reactions, their kinetics are hard to measure. That is the reason why numerous kinetic models neglect these secondary reactions.

In a recent study, Edeleva et al. studied the influence of secondary reactions on the kinetics of FRP of *n*-butyl acrylate (*n*BA) via a *k*MC model. It was demonstrated that the impact of the secondary reactions is pronounced already at intermediate temperature, and thus, radical polymerization reaction schemes should be detailed. Secondary reactions in the studied process contribute not only to the retardation of the overall polymerization kinetics but also alter the structure of the final polymeric molecules. The latter leads to the sufficient differences in the material properties between the polymer synthesized at low and high temperature.

Figure 4 (bottom left) also highlights the transition from FRP to CRP. In CRP, one can temporarily deactivate radicals in dormant species so that in the pool of primary reactions, one also has activation and deactivation. The bottom right of Figure 4 also depicts (secondary) chain transfer reactions that are unavoidable in any radical polymerization process, although under CRP conditions, they can be minimized. In what follows, an overview is given of relevant modeling contributions in which the reactions in Figure 4 are addressed [14,23,146,174,175,176,177,178,179,180,181,182,183].

#### 6.1.1. Initiation

Dossi et al. [184] investigated the initiation rate coefficients for four monomers that are relevant for industrial FRP (methyl acrylate, methyl methacrylate, acrylonitrile, and styrene) using five widely used initiators (azoisobutyronitrile, di-*tert*-butyl peroxide, potassium persulfate, 2,2-dimethoxy-2-phenylacetophenone, and dibenzoyl peroxide). The rate coefficients of the initiation reaction were obtained using DFT calculations. Upon comparing to the experimental values, the calculated rate coefficients are accurate enough despite the absence of solvation correction. The influence of the solvent was accounted for via an Evans–Polanyi–Semenov correction upon linking the calculated enthalpies with the experimental activation energies.

Another example of the application of QCC is the study of the self-initiation process performed by Srinivasan et al. [185,186,187], who developed the methodology for the theoretical investigation of 2-2′, 2′-4′, and 4-4′ cycloaddition reactions of monomers that are involved in the self-initiation mechanism. This work has been followed up by Lui et al. [188], who coupled DFT calculations with nonadiabatic TST to develop the methodology for the energy barrier calculation of the reaction involving spin cross-over. Eventually, Laki et al. [189] compared the experimentally derived self-initiation rate coefficients for acrylate monomers with the ones predicted theoretically. Experimentally, the process of radical self-initiation may compete with initiation with impurities, thus being difficult to measure. Consequently, QCC provide valuable insights into self-initiation. The data can be considered even more reliable than the experimental data, as the latter are prone to the influence of impurities, which are nearly impossible to avoid.

#### 6.1.2. Propagation (Non-Aqueous)

As propagation is the determining step in polymerization, accurate determination of the propagation rate coefficient is crucial. A number of experimental approaches exist to study the propagation reaction in radical polymerization; among them, pulsed laser polymerization with size-exclusion chromatography analysis (PLP-SEC) has been the most studied [190,191,192,193,194,195,196,197]. If an experiment is performed, the secondary reactions can influence the measurement of the selected propagation, leading to the inaccurate determination of the rate coefficient. In turn, computational studies of the propagation reaction are free from the impact of secondary reactions and thus can give important insights into the influence of the reaction conditions on the reactivity, e.g., solvent effects, pH, and hydrogen bonding.

Heuts et al. [69,70] predicted the absolute rate coefficients in FRP of ethylene with TST in the mid-1990s, accounting for the effect of internal rotations on the partition functions. In this contribution, the authors studied the chain-length dependence of the calculated propagation rate coefficient, showing that it converged by the hexyl radical stage. The accuracy of the prediction can be (insufficiently) increased further in case more advanced coupled internal rotation is considered, as developed by Van Speybroeck et al. [198,199]. Later on, Deglmann et al. [200] developed a methodology for the determination of propagation rate coefficients by the DFT method with the COSMO-RS solvation model. They benchmarked their theoretical results for most relevant monomers with the PLP-SEC data. The authors concluded that with the appropriate level of theory and suitable molecular models (up to 200 atoms), it is possible to predict the activation energies for propagation within 4 kJ/mol accuracy, which is a typical experimental error. Applying ab initio calculations with the COSMO solvation model, Izgorodina et al. [201] was able to achieve good agreement for the calculated and experimental values for acrylonitrile and vinyl chloride, even with small molecular models (e.g., 5 monomer units). However, upon studying the propagation of methyl acrylate and vinyl acetate, these authors pointed out that DFT methods for the gas-phase energies and simple continuum models for the solvation energies can lead to errors of several orders of magnitude. Therefore, such simplified strategies should be avoided for the general QCC of propagation reactions [202].

Theoretical investigation of the propagation reaction becomes specifically relevant if several propagation pathways are possible, e.g., head-to-tail and head-to-head monomer addition, or several types of propagating radicals exist simultaneously, such as for acrylates with end-chain radicals (ECR) and mid-chain radical (MCR) participating in the propagation. One can refer here to the work of Van Cauter et al. [203], who studied theoretically the head-to-head addition in vinyl chloride polymerization. The authors compared calculated and experimental relative sequences of head-to-head contents in polymers. They concluded that the ab initio calculations correctly predict this type of defect in the polymer structure.

The ab initio calculated propagation rate coefficients can also be inputted rate coefficients for *k*MC models in case of polymerization systems with a large number of reactive species. This has been demonstrated by Desmet et al. [66], who were able to model the PLP of vinyl acetate, inputting theoretical values of the reactive species.

Currently, acceptably accurate propagation rate coefficients have been reported for the monomers: styrene, methyl acrylate, butyl acrylate, ethyl acrylate, hydroxyethyl acrylate, methyl methacrylate, butyl methacrylate, glycidyl methacrylate, 2-hydroxyethyl methacrylate, vinyl acetate, vinylidene fluoride, hexafluoropropylene, and tetrafluorethylene [200,201,204,205,206,207,208,209,210,211,212,213,214,215].

Upon the calculation of propagation rate coefficients, one always has to explicitly consider the stereoselectivity of the propagation reaction. Tacticity control in FRP can be an important topic, since it affects the material properties of the polymers such as the mechanical strength, the melting and glass transition temperatures, or solubility. However, at first sight, most of the polymers produced by FRP lack tacticity and are mostly atactic polymers, since the propagation typically occurs through the sp^2^ planar radical species, inevitably leading to racemization. QCC calculation can be helpful to check this hypothesis. For example, Değirmenci et al. [207] reported a solvent effect for propagation with methyl methacrylate and the tacticity, which was related to the usage of methanol and (CF_3_)_3_COH as solvent. The formation of syndiotactic-rich polymer was the consequence of hydrogen bonding between the carbonyl oxygen of the monomer and alcohol’s hydrogen in the transition structures. The stabilizing effect of the (CF_3_)_3_COH on the transition structures has been attributed to the relatively higher steric effect of the solvent molecules and stronger hydrogen bond formation as compared to methanol as solvent. These results were obtained by application of PCM as the solvation model.

More examples of PCM application for QCC regarding the stereospecific propagation are given by Kayık et al. [216], who studied the radical polymerization of a series of 3 *N*,*N*-alkylacrylamide monomers. The authors were able to explain the favorable stereospecific addition modes by the interplay between the steric effects and the hydrogen bonding interactions. In another study by Özaltın et al. [217,218], the tacticity of N-isopropylacrylamide (NIPAAm) was modeled to understand the solvent effect via a combined explicit/implicit model. Such a sophisticated calculation technique provided the insight that the solvent either accelerates the propagation step by the stabilization of the transition state or enhances the tacticity by forming a complex that brings C=O and –NH groups to a closer proximity.

An interesting case is the FRP of ethylene. While the polymerization is carried out at high temperature and pressure (up to 350 °C and 3000 bar), it cannot be fully considered a gas phase process. Nevertheless, it is often studied via gas-phase computational tools. Heuts et al. [69] developed a method based on ab initio calculations and TST to access the kinetic rate coefficients for the ethylene polymerization. The authors highlighted the importance of the molecular model selection and suggested a hindered transition state in this reaction. Their results were in good agreement with the experimental data, suggesting a small influence of solvation when monomers are apolar. Konstantinov et al. [219] performed a computational study of the propagation rate coefficients and Arrhenius parameters for the ethylene FRP employing unimer, dimer, trimer, and tetramer models of the polymer chain. The calculations indicate that except for the unimer, little change occurs to the activation energy and pre-exponential factor as the system size increases. However, the direct comparison with the experimental results of Buback et al. [220] and Goto et al. [221] is not straightforward, as the activation volume needs to be calculated accurately to account for the pressure effects. However, the authors were able to achieve good agreement for the tetramer models. The theoretical calculations of the ethylene propagation have additional complexation of the reaction path degeneracy, four of which arise from the symmetry of the molecule. The overall reaction rate coefficient is the sum of the rate constants along all trajectories with the appropriate reaction path degeneracy. Konstantinov et al. [219] accounted for that as well, showing that about 30% of the total rate parameter is attributed to the “anti” transition state, which is higher in Gibbs free energy. The convergence of the k_p_ calculation results for molecular models larger than 4 is also pointed out by Van Cauter et al. [222].

Ethylene is typically copolymerized with other monomers; thus, the access to the reactivity ratios is important. Van Speybroeck et al. [223] screened several theoretical approaches to the calculation of the addition rate of methyl, ethyl, propyl, and butyl radicals to the ethylene molecule, which is an important step of copolymerization. Similarly, Filley et al. [224] used ab initio calculations to estimate the reactivity ratios in ethylene−vinyl acetate free-radical copolymerization. The authors found the small influence of the penultimate unit in this particular case.

#### 6.1.3. Propagation (Aqueous)

The solvent starts to play an important role in the mechanism of radical polymerization if some specific interactions between the monomer and the solvent exist. This is the case of for FRP in the aqueous phase. For the theoretical investigations of such systems, special attention should be paid to the modeling of the solvent–solute interactions via application of the solvation models. As an example, Thickett and Gilbert [214] applied PCM to the polymerization of acrylic acid in toluene and water. They obtained a lower activation energy for the polar aqueous medium, which is due to the better resonance stabilization of the transition state. Although a remarkably good agreement with experimental rate coefficients is reported in these contributions, it is clear that such approaches will not be able to describe, for example, monomer concentration effects in aqueous solution polymerization.

An overview of the potential of QCC for solvent and concentration dependencies of propagation rate coefficients for aqueous monomers was recently given by Deglmann et al. [200,225,226]. The authors claimed that the relative changes in rate upon transition from one medium were in good agreement with the experimental trends in cases where the COSMO-RS model was applied. Furthermore, with the COSMO-RS model, Kröger et al. [227] predicted the rate coefficients and reaction enthalpies of the propagation reactions in aqueous *N*-isopropylacrylamide/*N*,*N*′-methylenebisacrylamide and aqueous *N*-vinylcaprolactam/*N*,*N*′-methylenebisacrylamide systems with an accuracy of a factor of 2–10 compared to the experimental values. By analyzing the effect of rate coefficients on the microgel formation, the authors concluded that the differences in the magnitude of the propagation rate values are a reason for an inhomogeneous cross-linker distribution within the resulting microgel.

#### 6.1.4. Termination

An interesting application of QCC for termination reactions is the possibility of distinguishing between recombination and disproportionation. Indeed, if the termination occurs via recombination, the resulting polymer has the length of the sum of the two parent polymer chains, whereas in the disproportionation mechanism, the chain length of the “dead” polymer chains remains equal to those of the parent chains. Thus, the preferable termination mechanism affects significantly the number-average molar mass *M*_n_. As the structures of the products differ in case of termination by recombination and disproportionation, QCC can distinguish between these two pathways and provide the evidence for the preferable reaction to occur. In fact, some authors [228,229] point out that the transition states have different geometries for recombination and disproportionation.

A debate specifically exists for acrylic monomers. While it is commonly accepted that for acrylates, recombination is predominant, Bamford et al. [230] reported disproportionation to be the favorable pathway. Nakamura et al. [231,232] used both experimental methods and theoretical calculations to investigate the termination mode for the methyl acrylate monomer. The authors concluded that at ambient temperatures, disproportionation is the predominant mechanism.

#### 6.1.5. Secondary Reactions

The continuously increasing QCC potential allows the extension toward secondary reactions in radical polymerization. The investigation of such reactions can be motivated by two main reasons. The overall kinetics can be significantly influenced, and they can be the cause of deviations from the desired polymer properties and molecular structure. As shown in Figure 4, the reactions most relevant to FRP are the following: intermolecular hydrogen abstractions, backbiting, chain transfer to small molecules (solvent, monomer, or chain transfer agents), propagation of mid-chain radicals (MCRs) formed in backbiting, and β-scissions of these MCRs.

Theoretical investigation of the secondary reactions is obstructed by the absence of reliable experimental data for benchmarking for a broad monomer set. Nevertheless, QCC can provide useful information on the occurrence of a particular side reaction and the relative rate of it compared to main reactions. An example can be given by the work of Cuccato et al. [233], who studied the secondary reactions in FRP of *n*-butyl acrylate. For the backbiting reaction, the authors considered 1–3, 1–5, 1–7 and 7-3 possibilities. They concluded that 1–5 backbiting is favored, due to the stabilized 5-membered ring structure of the transition state. Furthermore, they concluded that 7-3 backbiting is also possible thus supporting the occurrence of radical migration in the FRP of *n*-butyl acrylate (at least under conditions with low monomer amounts). With the help of QCC, Yu et al. [234] provided the kinetic rate coefficient for the 1:5 backbiting reaction as well.

Backbiting and subsequent β-scission are also relevant for other monomers, as highlighted by Dossi et al. [235] for acrylonitrile. Using the 7-monomer unit models, the authors calculated the rate coefficients of backbiting and β-scission. The important finding here is that the authors suggest similar activation energies for β-scission to the “right” and to the “left” of the MCR, which is similar to acrylic monomers.

Another important reaction that contributes to the formation of the long-chain-branched polymers is chain transfer to polymer. QCC provides some insights into the kinetics of this reaction in case of alkyl acrylates, as presented by Moghadam et al. [236]. The abstraction of a hydrogen atom from a tertiary carbon atom was found to be the most favorable chain transfer to the polymer mechanism in alkyl acrylates. It is interesting to note that with the COSMO solvation model, the authors observed only a small influence of solvent on this reaction. Overall, it can be concluded that QCC has been applied for rate coefficients for secondary reactions with acrylic and methacrylic monomers [210,237], acrylonitrile, [235,238], and vinyl chloride [203,239,240].

#### 6.1.6. Controlled Radical Polymerization

QCC can give useful insights for the design of novel and more effective CRP-mediating agents, as they link the kinetic parameters to the (electronic) structure of the reactive species involved [241,242]. QCC have been successfully employed to identify structure–reactivity trends within series of mediating agents for RAFT polymerization, nitroxide-mediated polymerization (NMP), and atom transfer radical polymerization (ATRP). They enable assessing the suitability of the candidate-mediating agents for a given monomer, limiting experimental efforts. In case of some secondary reactions, structure–property correlations based on QCC can provide guidance for minimization of the side reactions’ impact as well. Hereafter, we showcase several examples of QCC applications for ATRP, RAFT polymerization, and NMP with particular focus on accounting for the solvent effects. Note that related to ATRP, so-called single electron transfer–living radical polymerization (SET-LRP) has been put forward as well [243,244]. The current work is limited to ATRP mechanisms in order to not overcomplicate the discussion.

The number of theoretical studies for RAFT polymerization is significant. For as long as this polymerization technique has been developed [245], QCC calculations have been employed to obtain mechanistic insights, reveal the factors influencing the addition-fragmentation equilibrium, as well as develop low computational cost screening procedures for the investigation of the RAFT agents [246,247,248,249,250,251]. QCC has specifically assisted in the identification of new fragmentation pathways in RAFT polymerization [252] and recently led to the first computer-designed RAFT agent [253,254].

As the mechanism of RAFT polymerization relies on the exchange between active and dormant species, the selection of the RAFT moieties is important for the synthesis of well-defined polymers [183,255,256,257,258]. The efficiency of RAFT agents is attributed to their transfer coefficients, which determine the interchange potential between dormant and living chains. Chemically, the selection of the Z and Y group in transfer agents (see Figure 4) is crucial to drive the polymerization success. For example, via two contributions, Rodríguez-Sanchez et al. [259,260] performed theoretical studies of the influence of the RAFT agent structure on the kinetic parameters. They pointed out that the reactivity is determined by both Z and Y groups, and they presented several RAFT structure-polymerizable monomer correlations. It has been further indicated that the Z group of RAFT agents determines which monomers they can adequately control. The more active dithioesters and trithiocarbonates control the polymerization of so-called “more activated monomers” (MAMs), whereas the less active xanthates and dithiocarbamates control the polymerization of “less-activated monomers” (LAMs) [261]. Thus, a drawback arises for poly-MAM-block-poly-LAM. In this context, high level QCC have predicted that a fluorine Z group (so-called F-RAFT agent) should provide good control over the polymerization of both MAMs and LAMs [253]. The fluorine Z group is predicted to destabilize the RAFT intermediate radical, promoting its fragmentation. At the same time, the reactivity of the thiocarbonyl group should not be affected significantly. Notably, some experimental studies confirmed the efficiency of these F-RAFT agents [254,262].

For completeness, it is mentioned here that an alternative approach for RAFT polymerization of LAMs and MAMs has been introduced by Bengalia et al. [263]. These authors proposed the application of protonable (so-called switchable) RAFT agents with a 4-pyridyl substituent. The protonation/deprotonation of the latter affect the stabilization of the RAFT intermediate radical, allowing the efficient polymerization of various monomers [264].

For NMP, QCC have also been applied. Specific focus has been on H-transfer reactions in the NMP of methacrylic monomers, the thermal instability of nitroxide, and reactions with oxygen. More in detail, one of the side reactions that lead to the loss of the NMP-controlled regime is the N-O bond cleavage in alkoxyamines. This process was observed experimentally for several types of nitroxides [265,266]. Upon investigating the homolysis of alkoxyamines by DFT, Gaudel-Siri et al. [267] noticed that N-OC bound homolysis can occur at elevated temperatures. However, it was observed that DFT showed large variations from the experimental bond dissociation energies (BDEs), although they were able to model the NO-C versus N-OC competition successfully. An extensive theoretical research of the N-OC bound cleavage in alkoxyamines was performed by Hodgson et al. [268], who investigated five- and six-membered cyclic alkoxyamines as well as linear ones. The authors accounted for the effect of the solvent by PCM and COSMO-RS models. The authors concluded that the N-OC bond cleavage is favored for the structures with the stabilized aminyl radical. Furthermore, the authors formulated an important methodological guidance for the theoretical investigation of such reactions with high accuracy.

Another example of QCC application for the investigation of secondary reactions is presented by Parkhomenko et al. [269], who performed an extensive investigation of intra-molecular rearrangement of model compounds to study the H-transfer reaction by DFT. The authors optimized the geometry of the transition state and obtained insights into the reaction mechanism, showing that the C-ON bond homolysis competes with intramolecular rearrangement. The experimental observation of the latter depends on the differences in the activation energies of the primary and side reaction. As the solvation model, the authors employed PCM, which allowed them to study the solvent effect as well. Gryn’ova et al. [270] used the high-level ab initio method in combination with COSMO-RS to model the solvent effects to calculate the reaction rate coefficients of the intermolecular H-transfer for five systems. The intramolecular reaction was shown to be kinetically disfavored. However, even its minor occurrence leads to unsaturated chain ends in the final polymer and decreases the living fraction. QCC assisted as well the development of the effective NMP agents with tunable reactivity [177,271,272,273].

QCC has been employed extensively for ATRP as well. ATRP has expanded significantly since its development [274,275,276], providing a wide range of catalytic systems, ligands, and experimental conditions. Nevertheless, copper-catalyzed ATRP (Cu-ATRP), i.e., the classical system, remains the most extensively applied and investigated one, as it allows polymerizing a wide range of monomers, using various initiators and solvents. Control of the polymer chain growth via Cu-ATRP is largely attributed to the activation/deactivation (pseudo-)equilibrium between [Cu^I^L]^+^ and [X–Cu^II^L]^+^ species, with L being a multidentate nitrogen-donor ligand and X being a halogen atom. The Cu^I^ species activates the dormant alkyl bromide chain end (see Figure 4, bottom left, with X=Br) to form a Cu^II^ complex and a propagating polymer radical. The latter propagates before it transfers the X atom from the Cu^II^ complex to reform a dormant species. For a polymerization being controlled and maintaining a reasonable rate, the ATRP catalyst system should have large activation and deactivation rate coefficients with the latter being the highest. These two coefficients depend on the structure of the halide, which is determined by the monomer being polymerized, and the structure of the ligand employed in the catalytic system.

For the ATRP-initiating systems, the strength of the C-X bond is important. Gillies et al. [277] employed DFT calculations to quantify the BDEs in a number of ATRP initiators and provided access to equilibrium coefficients. They observed good agreement between the calculated and experimental values. Lin et al. [206] researched the optimization of these initiators, taking into account the penultimate effect. The penultimate effect in ATRP is especially important for the activation–deactivation of the dormant species. Dormant species with more than one monomeric entity generally display a higher activation rate coefficient than the initiator. Lin et al. [206] investigated the magnitude of this effect on dimers involving methacrylate, methyl methacrylate, and propylene comonomers by calculating the BDE values. These authors observed that the penultimate effect was more pronounced in the methyl methacrylate systems. This means that dimeric initiators are much more efficient than the monomeric ones in the ATRP of MMA. In a later work, [278] the same group of authors screened many alkyl halides, employing the solvent corrections via PCM. The more advanced level of the calculations allowed for the more precise construction of the potential energy surfaces and unveiled the relevance of the primary and secondary reaction pathways.

The understanding of the effect of the ligands is also important for ATRP optimization. In this context, Tang et al. [279] and Fang et al. [280] recently provided structure–properties correlations. Finally, as in RAFT and NMP polymerization, QCC can be used to design highly efficient ATRP catalytic systems, optimize experimental conditions, and construct structure–properties correlations, as for instance exemplified by Woodruff et al. [281].

### 6.2. Case Study 2: (Metal Complex Catalyzed) Polymerization of Olefins

**Figure 5:**
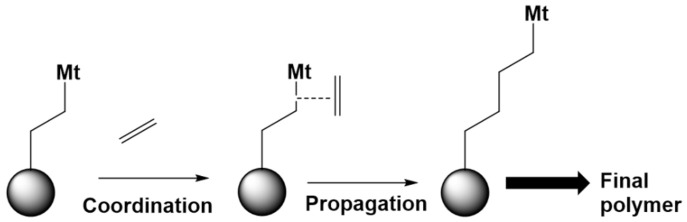
The main steps of catalytic olefin polymerization, as showcased with ethylene.

The core of QCC for polymerization lies in the prediction of the (intrinsic) reactivities. In the area of olefin catalytic polymerization, the main applications are in the assessment of reactivity for the classes of monomers and screening the activity of homogeneous and heterogeneous catalysts. The detailed advances in theoretical studies of catalytic polymerization are presented in the reviews of Ehm et al. [282] and Kang et al. [283]. A special case is the chain transfer-to-solvent reaction. The advances of the computational chemistry in that field have been recently reviewed by Zaccaria et al. [284]. In this subsection, for illustration purposes, a selected number of QCC examples are included for ionic polymerization with the special emphasis on solvent effects. A distinction is made between monomers and catalysts. The main steps of the catalytic olefin polymerization are shown in Figure 5.

#### 6.2.1. (Co)monomers

In a series of contributions by Kaufman et al. [285,286,287], QCC have been employed to investigate the mechanism of cyclic oxetane-based monomers. The reactivity in the cationic polymerization of these types of monomers is screened on the basis of the electrostatic molecular potential contour maps constructed via ab initio calculation with explicit accounting for a proton in the calculations. It has been found that the rate of cationic polymerization is proportional to the basicity, the base strength of the monomer, and the ring strain. The basicity was linked to the sizes of the negative regions on the map around the O atom. Experiments afterwards confirmed the relative reactivities of the theoretically studied monomers.

In a more recent study, Hlil et al. [288] addressed the reactivity of five- to eight-membered cyclic olefins in ring-opening metathesis polymerization with ruthenium catalysts, which are second-generation Hoveyda–Grubbs catalysts. They studied the polymerization in dichloromethane (DCM), toluene, and tetrahydrofuran (THF), applying DFT coupled with the SMD solvation model. The authors pointed out that all studied solvents reduce the energy of the intermediates and transition states equally.

It should be further put forward that the efficient copolymerization of ethylene with substituted polar monomers remains challenging due to the difference in the reactivity with typical catalysts. Chen et al. [289] address this problem via QCC investigation of the organoscandium-catalyzed ethylene + amino olefin copolymerization mechanism. PCM was used to account for the solvent effects. It was found that the copolymerization activity is largely governed by intermolecular amino olefin N-coordination with the catalyst.

#### 6.2.2. Homogeneous and Heterogeneous Catalysts

Catalyst development for ionic polymerization is one of the cutting-edge applications of QCC, as it allows estimating the catalyst reactivity prior to the actual synthesis. Furthermore, QCC provides guidance for the applicability of particular types of catalysts for the polymerization of functional monomers and for the model-guided synthesis of the catalysts with targeted properties.

With the help of DFT calculations, Vo et al. [290] determined for instance the initiation and propagation pathways of the polymerization reaction of isobutylene via a catalytic reaction with a Lewis acid catalyst (AlCl_3_) under aqueous conditions. The polymerization medium was explicitly accounted for by including two AlCl_3_ and one water molecule into the molecular model. Previously, it was assumed that AlCl_3_OH_2_ was the active species during the catalysis of those types of reactions, whereas Vo et al. [290,291] showed that the catalyst complex that is identified as the active species that catalyzes the initiation and propagation has the structure of a complex with two AlCl_3_ groups and one H_2_O group (AlCl_3_HOHAlCl_3_), producing highly acidic protons. The calculations are in good agreement with the experimental data, as a reaction rate comparable to the one using AlCl_3_HOHAlCl_3_ is observed.

Solvent effects are indeed important for the homogeneous catalytic systems, as pointed out by many researchers [292,293,294,295,296]. From this point of view, Belelli and Castellani [297] studied the Zr-based metallocene catalysts active sites employing PCM. The authors concluded that the PCM simulates the solvent effect with higher precision, providing a correct description of the catalytic system as the simulation results followed the observed experimental trends. Stability of the binuclear Zr based metallocenes was addressed by Meelua et al. [298]. The authors showcased that the stability of the binuclear catalytic complex is significantly affected by the solvent polarity through DFT calculations with the SMD solvation model. Thus, in order to conduct olefin polymerization or cationic ring-opening polymerization of polar cyclic monomers such as lactones and cyclic carbonates, the solvent should be selected carefully.

In turn, Castro et al. [299] highlighted that both solvent and dispersion effects need to be accounted for in the theoretical calculations for coordination polymerization. For the Zr-based catalysts, they showed that the solvent correction via SMD only leads to underestimation of the energetic barriers, whereas inclusion of solvent and dispersion correction provides good agreement between the theoretical and experimental results.

In addition, for the heterogeneous Ziegler–Natta catalytic systems with TiCl_4_ and its Lewis base complex on MgCl_2_ support, Cavallo et al. [300] studied the geometries of the active complexes and binding energies in propylene polymerization. DFT calculations with the COSMO solvation model provided structural information of the catalytic complexes, and relative energies between the different Ti complexes can be calculated with a reasonable accuracy. The crystalline structure of the catalysts and catalyst–support interactions are also addressed via QCC, even though the reaction medium does not play a significant role. Hence, the environment is typically calculated explicitly in those type of calculations. An example is here the work of Correa et al. [301], who explored the crystalline structure of TiCl_4_ catalysts on MgCl_2_ support.

The QCC results for metal–organic compounds should be treated with caution, as certain difficulties exist that arise from the treatment of the exchange-correlation in SE. Furthermore, the benchmarking of the calculated and experimental results is not always straightforward, as good agreement can be sometimes due to the cancellations of the errors. For the detailed summary of challenges existing in DFT for transition metal compounds, the reader is referred to the works of Harvey et al. [302,303,304].

### 6.3. Case Study 3: Step-Growth Polymerization

Polymers produced via a step-growth polymerization mechanism form an important class of materials with a broad application range. Among others, polyurethanes formed in the reaction of isocyanates with alcohols are an important class of polymer materials. If a urethane bond cleaves, a reversed to urethane formation reaction occurs, which can be considered as a possible production route for some important isocyanates. This reaction possesses a high energetic barrier, and the equilibrium is fully shifted toward the formation of polyurethanes. The addition of catalysts alters the rate of urethane bond formation, thus facilitating the polymerization process. Furthermore, a number of side reactions may occur in isocyanate–alcohol systems, forming by-products.

As the mechanism of polyurethane formation is rather complex, it is not surprising that computational studies can assist in the identification of acceptable reaction pathways. For instance, one of the questions that has arisen is the mechanism of alcohol addition to the isocyanate bond, as it can happen either through C=N or C=O bonds. In what follows, some key QCC contributions are discussed that can be seen in a general context for other step-growth polymerizations as well.

More in detail, with the help of DFT calculations and the PCM solvation model, Cysewski et al. [305] studied the non-catalytic two-step mechanism with addition of the hydroxy group of the alcohol molecule to the C=O bond of the isocyanate. They calculated the activation energies of urethane formation in benzene and identified the structures of the transition states and intermediates. Despite the high level of theory, only qualitative agreement with the experimental data was achieved.

A series of studies by Samuilov et al. [306,307,308] addressed the mechanism of the isocyanate–alcohol reaction for a number of isocyanates and alcohols via QCC with the PCM solvation model. These reactions were considered to proceed via four-membered ring transition states. The results clearly show that the addition of the alcohol hydroxy group to the C=N bond is favored compared to the addition to the C=O bond. The autocatalytic role of the alcohol was pointed out as well. The mechanism of alcohol molecule addition to the isocyanate bond is also addressed by Çoban and Konuklar [309], who computed free energy profiles of bimolecular urethane formation in benzene based with PCM, and by Raspoet et al. [310], who used the HF method for the calculation of the transition states, also employing PCM. All authors agreed that the addition of a hydroxy group to the C=N was more preferable and showed the importance of autocatalysis. Application of the hybrid SMD model by Cheikh et al. [311] showed that isocyanates may also participate in the autocatalytic pathway for urethane formation.

**Figure 6:**
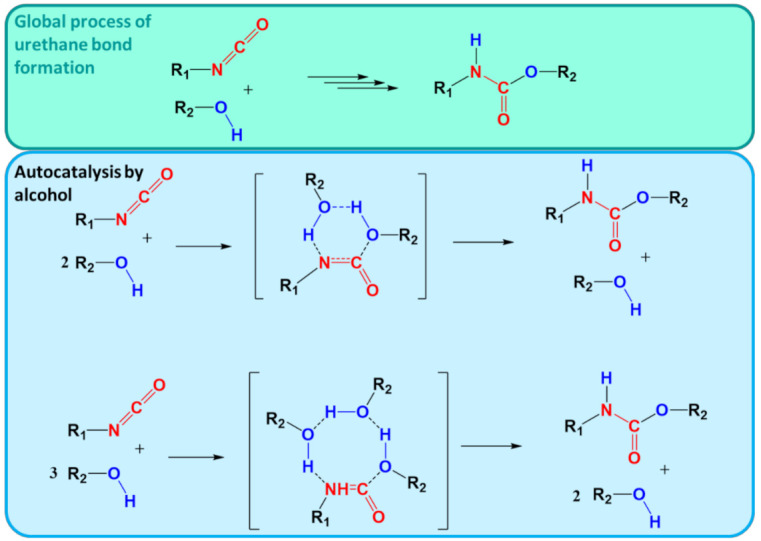
Global reaction of urethane bond formation being the basis of polyurethane step-growth polymerization. Mechanism of autocatalysis by alcohol molecules as explored by Gertig et al. [312].

Gertig et al. [312] performed a computational study of urethane polymerization/depolymerization coupled with the experimental study of urethane cleavage for non-catalytic systems, assuming the reaction mechanisms presented in the literature. As the authors aimed to study a broad range of reaction conditions, i.e., temperature, alcohol concentration, and reaction medium, they employed the COSMO-RS solvation model for a higher accuracy. The main goal was to evaluate the role of autocatalysis in the polyurethane formation. The authors identified various transition states for the non-catalytic reaction as well as for autocatalysis by the alcohol, showing quantitative agreement with the experimental literature data with reasonable accuracy. They identified two main reaction pathways in the autocatalytic route, i.e., autocatalysis by one and by two additional alcohol molecules, corresponding to the reaction via transition states with six- and eight-member ring structures. The latter seemed more important in non-polar media and at high alcohol concentrations, whereas the reaction via transition states with six-ring structures contributes strongly occurs in polar solvents and at low alcohol concentrations. The contribution of the completely non-catalytic reaction to the overall observed rate of urethane formation turned out to be negligible. It was also shown that for the depolymerization reaction, autocatalysis also plays an important role. The mechanisms of the autocatalytic urethane bond formation are presented in Figure 6.

### 6.4. Case Study 4: Polymer Environmental or Aging Degradation

**Figure 7:**
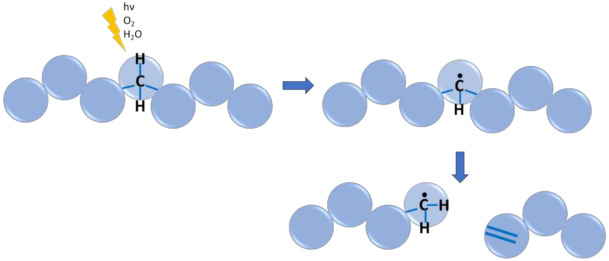
Schematic representation of polymer degradation under environmental conditions starting from H-atom abstraction with consecutive polymer chain β-scission.

Due to light, heat, humidity, air, or a combination of those, many polymers undergo degradation [313,314,315,316,317,318,319,320,321], as also shown in Figure 7. It can be either an undesirable process that alters the polymer processing and causes the decrease in the (average) molar mass, or it can be a targeted process if polymer recycling through depolymerization is considered.

Depending on the chemical structure of the polymer, different reactions can contribute to the polymer degradation. For instance, if polyesters are considered, the β-scission reaction at the ester linkage is the preferable mechanism. For polyolefins, radical degradation is the preferable mechanism. The basic scheme for polymers in the presence of oxygen, i.e., autooxidation, was originally developed by Bolland et al. [322,323,324] for rubbers and lipids. According to their scheme, the reaction of hydrogen abstraction from the polymer by the peroxyl radical (ROO˙ + RH →ROOH + R˙) is the main event. However, recently, Gryn’ova et al. [325] showed via QCC that this reaction has a large energy barrier (10–65 kJ mol^−1^), which cannot be lowered neither by elevated temperature nor solvation. To account for the effect of the solvent, the authors used the COSMO-RS model. In fact, they observed lower BDEs in solution than in the gas phase. Furthermore, the authors showed that structural defects are responsible for the autooxidation for most polyesters and most polyalkenes. These defects, such as terminal or internal double bonds, are formed either during polymerization or in the degradation process itself. Based on the main reaction step in the degradation process, the authors proposed several mitigation strategies for enhancement of the polymer stability.

Another important QCC application for the prediction of the stability and degradation mechanisms is the work of Okanishi et al. [326] focusing on polymer electrolyte fuel cells, as the insight for the enhancement of the membrane stability should facilitate the commercialization of these types of power sources. A typical membrane in a polymer electrolyte fuel cell consists of polymers with a fluorinated backbone, preferably perfluorosulfonic acid (PFSA) polymers, modified with sulfonic groups that facilitate the transport of protons [326,327,328,329]. The degradation process can occur due to mechanical, thermal, and chemical impact. Generally, it is assumed that the chemical degradation of a PFSA membrane is caused by the attack of free radicals from hydrogen peroxide (H_2_O_2_), which is formed naturally in the atmosphere. In order to find the degradation initiation step, the BDE for the various bonds in the PFSA polymer’s structure was calculated [330,331,332,333,334,335,336,337,338,339]. On the basis of that, the unzipping from the chain end mechanism was confirmed [340,341]. Interaction with solvent also plays an important role for the PFSA membranes, as they change the conductivity.

Furthermore, to study the degradation mechanism of PFSA under the attack of OH radicals, Yu et al. [342] employed the BP model to correctly take into account the charged structures. The authors concluded that OH radicals attack the side chain of the polymer with the formation of sulfuric acid. The findings of Yu et al. are in good agreement with experimental observations of the degradation products by ^19^F NMR and pH decrease [343]. The PCM model was also successfully applied by Panchenko [344] for the theoretical investigation of the degradation mechanism for sulfonated polyether(ether)ketone (sPEEK) and polyethersulfone (PSU), which are also promising materials for fuel cell construction. The author proposed a mechanism of OH radical addition to the aromatic rings of the polymer.

Another example of PCM solvation model application is the investigation of poly(3-hexylthiophene) polymer degradation in organic photovoltaics [345]. The calculations were performed for the polymers in a solid state. As these types of polymers are exposed to intensive UV irradiation, their stability is crucial for the performance. Sai et al. concluded that in the condensed phase, the degradation process starts with the radical attack on the polymer side chain. The authors underlined that the application of the solvation model is important for the correct conclusion, as the correction to the solid state (as a condensed phase) completely changed the final calculation results.

It is interesting to note that application of the implicit solvation model did not affect much the conclusions driven for the gas phase QCC made by Ebadi et al. [346] for the degradation process of PEO, PVA, PEC, PTMC, PCL, polyethylenimine (PEI), and polyacrylonitrile (PAN) host polymers in polymer/lithium batteries with proposed decomposition pathways through C_carbonyl_–O_ethereal_ and C_ethereal_–O_ethereal_ bond cleavage. In fact, the authors obtained reasonable results of the charged structures already with a simple PCM model. This was also showcased by Xing et al. [347], who studied the stability of the propylene carbonate under oxidative decomposition in the presence of anions in lithium ion batteries. Anyway, in general, the PB model is more applicable if ionic structures are considered. This was pointed out by Ma et al. [348], who addressed the stability of polyacrylamide (PAM)-based mining fluids toward degradation in saline for low pH solutions. Thanks to the advanced calculation method, it could be concluded that both base- and acid-catalyzed reactions have transition barriers in the range of 20–30 kcal mol^−1^, which are much lower than the uncatalyzed reactions (40–50 kcal mol^−1^) under the neutral condition. The calculation results support the experimental observation of enhanced PAM hydrolysis in the presence of salts.

### 6.5. Case Study 5: Material Properties

**Figure 8:**
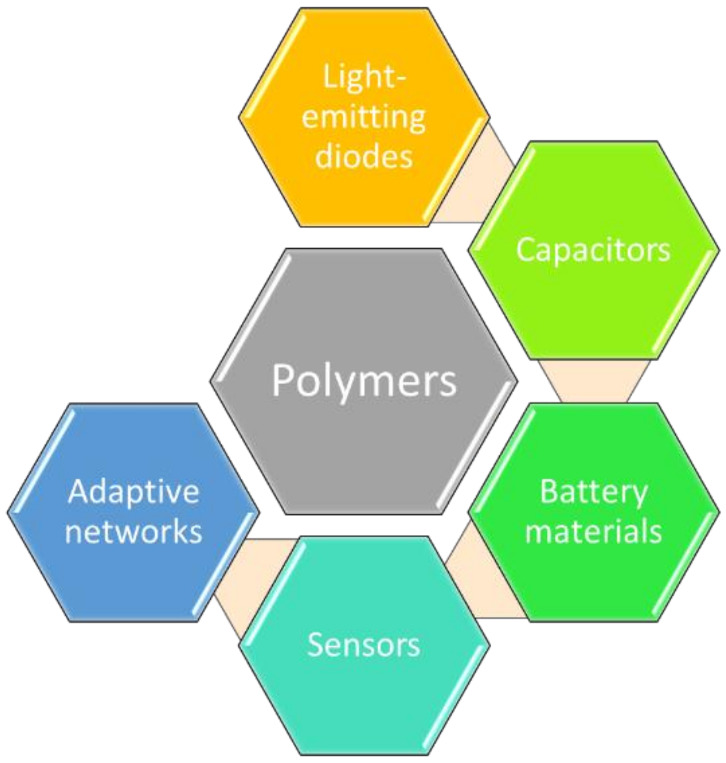
Application of polymers in electronic devices.

QCC are primary applicable to the molecular level and then translated to study kinetics and thus time dependencies. In some cases, this kinetic translation step is avoided, and the “basic” QCC results can provide direct evidence of the material properties. To make the reader aware of this QCC potential in the present subsection, emphasis is placed on the examples of polymer batteries and capacitors, although polymers find a broad range of application in microelectronics, as depicted in Figure 8 [349,350].

#### 6.5.1. Polymer Batteries

In the present section, focus is first on Li-ion batteries (LIBs) and then on electrode developments, all in view of highlighting QCC potential. LIBs with non-aqueous liquid electrolytes have made great advances in energy storage over the past few decades. Lithium metal batteries (LMBs) pose even superior properties, as the development of solid polymer electrolytes (SPEs) enhances their safety and energy density. The main components of the battery element are the cathode, anode, and electrolyte. Extensive theoretical and experimental investigations were carried out for understanding the chemical and physical nature of the electrochemical processes taking place during charge/discharge processes in batteries, thus contributing to the enhanced performance of the energy storage devices.

He et al. [351] specifically gave a general overview of the QCC development in the field of battery materials. Allam et al. [352] reviewed machine learning DFT-based methods for the development of electrode materials in LIBs. Yan et al. [353] focused their review on the development of cathode materials and the QCC relevance. Wang et al. [354] overviewed the research in anode solid electrolyte interphase modeling. A step forward in the energy storage device development is the lithium–sulfur (Li-S) batteries development. Furthermore, Yang et al. [355] reviewed the state-of-the-art in Li-S energy storage devices enhancement. The computational studies of carbon nitride-based materials of energy storage applications were discussed in the review by Adekoya et al. [356]. Considering the interaction of battery molecules with the medium, in his review, Leung [357] pointed out that the common method should be explicit modeling.

This QCC potential also follows from electrode design. The electrodes consist of a radical polymer moiety in the conductive medium. Typically, the polymer contains nitroxyl radical substituents that may easily undergo reversible oxidation, forming an oxoammonium cation, or reversible reduction, forming an aminoxyl anion. As the performance of the energy storage device depends on the above-mentioned electrochemical reaction, certain optimization can be achieved via QCC. For instance, the methodology of redox potential calculation is given in the paper of Moens et al. [358]. These authors put forward the importance of the solvation model for accurate calculations, as exemplified by the calculation of redox potentials with the PCM solvation model.

The methodology and accuracy of DFT/PCM calculation of nitroxides’ properties was revised by Tanaka et al. [359]. The accuracy of the QCC toward the prediction of the redox potentials of the radical substituted polymers was also addressed by Dardenne et al. [360], who applied DFT with the SMD solvation model. The authors concluded this method to be accurate and computationally less demanding so that it can be used for the prediction of the organic polymer batteries. The mentioned approach (high-throughout DFT/SMD modeling) was successfully applied by Cruz et al. [361] for phenazines as well, which are a new class of organic compounds for electrochemical energy storage applications. The authors screened 200 phenazine derivatives with electron-donating or -withdrawing substituents at different positions in non-aqueous media. They concluded that depending on the substituents and their position in the phenazine structure, the redox potential can be varied within a significant range.

#### 6.5.2. Capacitors

To improve the electrical energy storage devices’ performance, e.g., capacitive energy density, breakdown strength, and dielectric constant, research has been aimed at exploring new polymer nanocomposites, ferroelectric crystalline, or amorphous polar polymers.

For example, as shown by Ma et al. [362], polythiourea can be one of those promising materials. In order to enhance the processability, some functional moieties are introduced into the polymer backbone. DFT studies were used in the rational design of polythioureas containing different chain segments. Then, further computational studies were applied to calculate and compare electronic and dielectric properties. Lastly, a series of polymers were actually synthesized and investigated in terms of dielectric constant and loss, band gap, charge–discharge behavior, and DC breakdown strength. In order to increase the accuracy of the calculations and identify the three-dimensional structure of the polymers, the authors explicitly accounted for van der Waals interactions.

Screening of the materials for the anode can also be facilitated with QCC. Cui et al. [363] tested a polymer from polyarylimide and porphyrin as a new anode material for the aqueous zinc-ion hybrid capacitors (ZIHCs). Via DFT, they concluded that the Zn ion storage on the porphyrin nitrogen sites of the polymers is irreversible. However, they can increase the conductivity of the polymer and enhance the storage capacity of the device. This finding was consistent with the performed electrochemical tests.

The most important part of capacitors is the dielectric medium, which can be polymer-based. The strategies of polymer-based dielectric research via QCC are reviewed by Wang et al. [364]. In this work, focus is on the typical DFT calculations used to determine the properties of dielectrics, such as the dielectric constant and band gap by Wang et al. [365]. Nowadays, biaxially oriented polypropylene (PP) is the most common dielectric used for high-energy-density capacitors, but the dielectric constant is low. Furthermore, the properties of PP may be enhanced via introducing the OH groups. Therefore, DFT calculations are used to determine the effect of OH-functionalized PP and trapped moisture inside the dielectric. As the authors aimed to simulate the oriented polymer, they selected two “chains” arranged in head-to-tail configuration, thus accounting for the medium explicitly. Furthermore, one to two water molecules were added to the molecular system. The average value of the total dielectric constant increased for the polymer and polymer–water complexes upon comparison with the pure polymer. For PP, the total dielectric constant could be completely attributed to electronic contributions, but the increase due to the addition of OH groups could be linked to ionic contributions. Adding two H_2_O molecules leads to the formation of an H-bonded ring containing two OH groups and the two H_2_O molecules. It is important to mention that DFT thus clarifies the effect of the OH group and the addition of H_2_O, but it does not take into account the morphological variations of PP if functionalized with OH groups.

DFT calculations can also be used to obtain the band gap of different types of dielectrics. For instance, research has been done to identify the relationship between the electronic, ionic, and total dielectric constant with the band gap by Gonze et al. [366,367]. An inverse relationship has been found between the electronic dielectric constant and the band gap. This can be understood because the electric part of the dielectric response is a sum over electronic transitions from occupied to unoccupied states. No relationship has been found between the ionic dielectric constant and the band, as shown by Pilania et al. [368,369]. Thus, the total dielectric constant can be increased by increasing the ionic dielectric constant without compromising the band gap.

## 7. Conclusions

QCC is becoming more and more a strong tool to support kinetic modeling studies. With the development of novel software packages [370,371] and computational facilities, QCC has become a standard tool for organic and polymer chemists and engineers. In the present contribution, this has been specifically illustrated for polymerization (kinetics). In the polymerization field, one needs the appropriate QCC overall methodology, focusing on the correct oligomer approximation and the suited solvation model. Here, one can distinguish between implicit, explicit, and hybrid models that all have their advantages and disadvantages but jointly have allowed improving the mechanistic understanding of polymerization reactions in the condensed phase.

Ideally, one inputs absolute data on rate coefficients via TST in larger-scale kinetic models. Corrections for diffusional limitations due to viscosity increases are likely needed, and during scale-up, macro-scale modeling is inevitable. The current work highlights that in the following decades, much more advanced multiscale modeling tools will be available, as a better embedding of the molecular scale will be in reach. With more appropriate solvation models, one can for instance also study better dispersed phase polymerization in which in each phase reactive species will have different affinity toward further chemical modification.

For chemistries in which QCC rate coefficients are less accurate, preference should be given to the understanding of relative trends. In any case, both step- and chain-growth mechanisms can be considered, as illustrated through various case studies.

It should be further realized that (intrinsic) rate coefficients are essential input parameters in kinetic models, with often the need of such coefficients for both main and side reactions. The longer the model polymer chain, the more realistic the model, but the more difficult it will be to obtain accurate and robust thermodynamic and rate parameters: the many degrees of freedom regarding internal rotation and tacticity demand a large number of calculations, and the entropy is more difficult to predict, particularly in the liquid phase.

In addition, new trends are expected. While automated structure prediction algorithms exist and might assist in this regard, they never gained broad popularity, which was often due to a combination of the large computational power required, limited chemistry for which it was designed, and user input/evaluation that was still required anyhow. However, recent advances in neural network development might assist to reduce computational demands in the prediction of solvation energies, solution-phase thermodynamics, and minimum-energy structures. A more versatile implementation of multiscale methods could in this context lead to the simulation of longer polymer chains for which kinetics can still be predicted accurately.

## Figures and Tables

**Table 1:** Comparison of explicit and implicit solvent models for GCC.

	Explicit Models	Implicit Models
Features	All solvent molecules are explicitly represented.	Representation of solvent as a continuum.
Advantages	Realistic physical system description. Detailed interaction modeling is provided. High accuracy of calculation. Full details on the molecular geometry.	Simple. Low computational costs No explicit solvent atoms. High level of theory is possible due to low computational costs.
Disadvantages	Expensive for computation. Long iterative cycle required to equilibrate solvent to solute. Often solvent and solute are not polarizable. Large fluctuations in calculation results due to use of small system size.	Ignoring of specific short-range effects. Less accurate. Need to define an artificial boundary between the solute and solvent. No “good” model for treating short-range effects (dispersion and cavity).
